# Structural Configuration Effects of Freestanding TiO_2_ Nanotube Arrays on Power Conversion Efficiency in Dye-Sensitized Solar Cells

**DOI:** 10.3390/ma18225101

**Published:** 2025-11-10

**Authors:** Gangasagar Sharma Gaudel, Seung-Ju Yu, Hwa-Young Yang, Ye-Chong Moon, Sang Hoon Kim, Sang-Ho Park, Bong-Hyun Jun, Young Jun Kim, Won-Yeop Rho

**Affiliations:** 1 Graduate School of Integrated Energy-AI, Jeonbuk National University, Jeonju-si 54896, Republic of Korea; gangasagarsg@gmail.com (G.S.G.); ysj__0708@naver.com (S.-J.Y.); catalystm47@gmail.com (Y.-C.M.); 2 School of Energy and Chemical Engineering, Ulsan National Institute of Science and Technology (UNIST), Ulsan 44919, Republic of Korea; hyang@unist.ac.kr; 3 JCHI GLOBAL Co., Ltd., Incheon 22004, Republic of Korea; 4 School of Chemical Engineering, Jeonbuk National University, Jeonju-si 54896, Republic of Korea; 5 Department of Bioscience and Biotechnology, Konkuk University, Seoul 05029, Republic of Korea; 6 Environmental Safety Group, Korea Institute of Science and Technology Europe (KIST-Europe), 66123 Saarbrücken, Germany; 7 School of International Engineering and Science, Jeonbuk National University, Jeonju-si 54896, Republic of Korea

**Keywords:** dye sensitized solar cells, anodization, freestanding TiO_2_ nanotube arrays, configuration

## Abstract

**Highlights:**

**What are the main findings?**

- Four f-TNA structural configurations (closed/open, up/down) were compared to optimize DSSC performance.
- The **open** f-TNA structure, with the barrier layer removed, showed the highest power conversion efficiency (PCE ≈ 7.73%).
- The best PCE (7.73%) was achieved using the **open-up** f-TNA photoanode configuration. Barrier layer removal in f-TNA effectively minimized charge transfer resistances (*R_ct1_* and *R*_*ct*2_).
- The closed-up configuration yielded the lowest PCE (5.52%) due to poor electrolyte diffusion kinetics.

**What are the implications of the main findings?**

- The structural configuration and orientation of f-TNA are critical design parameters for high-performance DSSC.
- Structural optimization alone is a highly effective, additive-free strategy for maxim-izing TiO_2_ nanotube DSSC efficiency.
- Opening the f-TNA bottom is essential for enhancing charge collection and improv-ing electrolyte penetration.
- The study presents fundamental guidelines for engineering f-TNA electrodes in next-generation photoelectrochemical devices.

**Abstract:**

Dye-sensitized solar cells (DSSCs) are known for their excellent low-light performance, cost-effectiveness, and flexibility. The photoanode has a crucial role in enhancing the overall performance of DSSCs and can be modified with different nanostructures. This study explores the impact of photoanode structure on the power conversion efficiency (PCE) of DSSCs, where four configurations of freestanding TiO_2_ nanotube arrays (f-TNAs), closed-up, closed-down, open-up, and open-down, were employed as photoanodes. Performance was evaluated based on current density (*J_sc_*), open-circuit voltage (*V_oc_*), fill factor (FF), and PCE concerning dye adsorption, electrolyte diffusion, electron transport, and barrier layer. DSSCs based on open configurations, open-up and open-down f-TNAs, demonstrated superior performance, achieving PCE of 7.73% and 7.71%, respectively. The primary distinction between the DSSCs based on open-up f-TNAs and those based on open-down f-TNAs lies in the dye adsorption time and electron diffusion characteristics. The PCE for DSSCs with closed-down f-TNAs was measured at 6.78%, while DSSCs with closed-up f-TNAs showed a lower PCE of 5.52%. The presence of a barrier layer under the bottom of f-TNAs impacted the PCE for DSSCs with closed-down f-TNAs, whereas for DSSCs with closed-up f-TNAs, insufficient dye loading, poor electrolyte diffusion and barrier layer reduced the performance.

## 1. Introduction

The increasing population and rapid industrialization are escalating energy demand, leading to the production of various energy sources. The dependence on non-renewable energy has raised significant environmental concerns, including the depletion of fossil fuels and the worsening of climate change [1]. Consequently, researchers are concentrating their research efforts on alternative green energy solutions [2]. Solar energy has emerged as a crucial area of research due to its sustainability and immense potential [3].

The solar industry has focused on developing highly efficient, long-term stable, and cost-effective photovoltaic (PV) technologies. PV cells, commonly known as solar cells, convert light energy into electrical energy through the photoelectric effect [4]. To date, several types of solar cells have emerged, including silicon solar cells [5], perovskite solar cells (PSCs) [6], organic solar cells (OSCs) [7], quantum dot solar cells (QDSCs) [8], and dye-sensitized solar cells (DSSCs) [9]. Most solar cells have complex fabrication processes requiring expensive infrastructure, which has hindered further efficiency and sustainability improvements. However, DSSCs offer promising costs and ease of manufacturing alternatives [10]. DSSCs are recognized for their simple and low-cost production methods, light tunability, semitransparency, and ability to perform efficiently even in cloudy conditions [11].

Since O’Regan and Grätzel’s initial report in 1991 [9], the power conversion efficiency (PCE) of dye-sensitized solar cells (DSSCs) has significantly increased, now exceeding 15% [12]. The DSSC industry has committed to ongoing efforts to enhance efficiency and has attracted growing research interest through the engineering of materials, layers, and architectures. A typical DSSC has a sandwich configuration consisting of a photoanode, dye as a sensitizer, an electrolyte, and a counter electrode (CE) [13]. Advances in materials for sensitizers, electrolytes, CEs, and particularly photoanodes have greatly improved the performance of DSSCs. Enhancements can be achieved by modifying dye properties to broaden optical absorption, optimizing interfaces to minimize charge recombination, and aligning energy levels between dyes and semiconductor films [14]. The photoanode plays a crucial role in electron transport and is one of the most extensively studied components of DSSCs [15]. Furthermore, diffusion resistance at the photoanode has a significant impact on the overall performance of the solar cell [14].

Nanomaterials used as photoanodes have significant potential to enhance electron transport and enable higher dye loading by optimizing band alignment, morphology, dye adsorption, and optical properties [16]. Various metal oxide semiconductors, including zinc oxide (ZnO) [17], titanium dioxide (TiO_2_) [18], and tin oxide (SnO_2_) [19], have been investigated as photoanode materials for DSSCs. Among these, TiO_2_ is particularly notable as one of the most extensively studied photoanode materials due to its high chemical and thermal stability, low cost, high refractive index, and favorable conduction band, all of which facilitate efficient electron injection from sensitizers [20,21,22]. These challenges can be mitigated by utilizing one-dimensional (1D) nanostructures like nanotubes, nanowires, and nanorods [23]. However, despite the advantages of TiO_2_ nanoparticles, challenges such as charge recombination, limited electron diffusion length, and electrolyte diffusion issues still need to be addressed. These challenges can be mitigated by utilizing one-dimensional (1D) nanostructures like nanotubes, nanowires, and nanorods [24]. The performance of DSSCs is greatly influenced by the physical and structural properties of TiO_2_, including crystal size, morphology, phase composition, porosity, thickness of the TiO_2_ layer, and specific surface area [21,25]. These structures have gained attention due to their direct connection of photogeneration of electrons/holes [26] with the charge collection electrode, as well as improved dye adsorption, ultimately enhancing cell performance.

The preparation of a TiO_2_ nanostructure presents challenges, and adjusting parameters such as growth time, growth temperature, starting reactant concentration, acidity, and additives can influence the diameter, length, and density of the structures [27]. Complex morphologies of TiO_2_ nanotubes can be synthesized using anodization and hydrothermal methods [28,29]. TiO_2_ nanotubes produced through the hydrothermal method tend to have random orientations [30] and present difficulties in achieving consistent size and uniformity. TiO_2_ nanotubes produced through anodization offer precise control over dimensions and result in highly ordered, vertically aligned arrays with improved uniformity, which are referred to as TiO_2_ nanotube arrays (TNAs) [18]. However, the performance enhancement of DSSCs utilizing TNAs encounters obstacles such as limited surface area, which impacts dye loading and light absorption, as well as poor electron transfer due to high internal resistance and charge carrier trapping that impede current generation due to the barrier layer under the bottom of the TNAs. Additionally, while the outer structure of TNAs is cylindrical, the inner structure is conical, meaning TNAs have distinct configurations.

This study investigates how the structural configuration of freestanding TiO_2_ nanotube arrays (f-TNAs) influences the power conversion efficiency in DSSCs. TNAs produced through anodization were separated from the Ti plate to form f-TNAs, and the barrier layer under the bottom of the f-TNAs was removed and made open using an etching process to investigate the influence of the barrier layer on DSSC performance. We examined the impact of parameters such as current density (*J_sc_*), open-circuit voltage (*V_oc_*), fill factor (FF), and power conversion efficiency (PCE) on the configurations of TNAs in DSSCs. Our findings provide insights into how the orientation of the nanotube and variations in nanotube length, wall thickness, and surface area impact the photovoltaic performance.

## 2. Materials and Methods

### 2.1. Preparation of Freestanding TiO_2_ Nanotube Arrays (f-TNAs)

The synthesis of TiO_2_ nanotube arrays (TNAs) was accomplished through a two-step anodization process. Initially, a thin titanium plate (Ti, 99.7% purity, Alfa Aesar, Heysham, UK, 2.5 cm × 4.0 cm × 100 μm) served as the substrate for the first anodization. The 1st anodization took place in an electrolyte solution comprising 0.8 wt% NH_4_F (Sigma-Aldrich, Milwaukee, WI, USA) and 2 vol% H_2_O in ethylene glycol (Daesung Petrochemical Co, Yeosu, Republic of Korea), maintained at 25 °C under a constant 60 V DC voltage.

To enhance crystallinity, the resulting TNAs on the Ti plate were annealed at 500 °C for 1 h in ambient air. Subsequently, to facilitate TNA separation, the 2nd anodization was accomplished. The 2nd anodization utilized the same electrolyte solution but applied a lower voltage of 30 V DC. Following the 2nd anodization, the Ti plate was immersed in a 10% H_2_O_2_ (Sigma-Aldrich) solution. Finally, to remove the barrier layer at the bottom of the TNAs, the TNAs were subjected to ion milling via Ar^+^ bombardment (beam acceleration voltage: 1000 eV, current density: 1 mA/cm^2^, duration: 90 min, incidence angle: 45°). The TNAs were then sintered for 1 h to remove the residue ions and heal possible milling-induced defects.

### 2.2. Fabrication of DSSCs

The structure of the DSSCs comprised a transparent conductive oxide (TCO), TiO_2_ compact layer, TiO_2_ nanoparticle layer, freestanding TiO_2_ nanotube arrays (f-TNAs), dye, electrolyte, Pt counter electrode, and a second TCO layer. To fabricate the device, a TiO_2_ compact layer was first deposited onto fluorine-doped tin oxide (FTO) glass (Solaronix SA, Aubonne, Switzerland) by spin-coating a 5 wt% solution (Sigma-Aldrich) of titanium di-isopropoxide bis(acetylacetonate) in butanol, followed by thermal annealing at 500 °C for 30 min in air. A TiO_2_ nanoparticle layer was subsequently applied to the compact layer using the doctor blade technique, utilizing commercial TiO_2_ paste. The TiO_2_ nanoparticle layer had a final thickness of approximately 3 μm after annealing.

During this step, f-TNAs were simultaneously integrated into the nanoparticle layer to ensure close electrical and mechanical contact. For optimal interfacial adhesion between the TiO_2_ nanoparticles and the f-TNAs, the substrate was annealed again at 500 °C for 1 h under ambient conditions. The f-TNAs were then treated with a TiCl_4_ solution (Sigma-Aldrich) to improve surface properties and electron transport.

The f-TNAs (0.5 cm × 0.5 cm) on FTO glass were immersed in a dye ((Bu_4_N)_2_Ru(dobpyH)_2_(NCS)_2_, N719, Solaronix) solution (Solaronix SA) at 50 °C for 8 h for sensitization. The sensitized electrodes were assembled with a Pt counter electrode, using a 60 μm-thick Surlyn^®^ (DuPont, Wilmington, DE, USA) hot-melt spacer as a gasket. Cell sealing was performed by pressing and heating at 120 °C for 10 min to ensure a robust and airtight device. Electrolyte was introduced into the inter-electrode space by capillary action. The electrolyte consisted of 0.7 M 1-butyl-3-methyl-imidazolium iodide (BMII), 0.03 M I_2_, 0.1 M guanidinium thiocyanate (GSCN), and 0.5 M 4-tert-butylpyridine (TBP) dissolved in anhydrous acetonitrile/valeronitrile (85:15 *v/v*, purity > 99.5%).

All solvents and electrolyte reagents were prepared and stored under controlled humidity conditions (<40%) to minimize water content and ensure purity. The binary solvent mixture was selected based on literature standards for optimal viscosity and ionic mobility (ca. 0.9–1.2 mPa·s at room temperature). The Pt counter electrode was produced by drop-casting 0.5 mM H_2_PtCl_6_ in ethanol onto FTO glass, followed by thermal annealing at 400 °C for 30 min. The catalytic activity of the Pt layer toward the I^−^/I_3_^−^ redox couple was confirmed by cyclic voltammetry prior to assembly.

### 2.3. Characterization

The current density–voltage (*J–V*) characteristics of the dye-sensitized solar cells (DSSCs) were evaluated using a KEITHLEY 2400 electrometer under simulated AM 1.5 illumination (100 mW/cm^2^). The light source consisted of a 1 kW xenon lamp with an AM 1.5 filter (PEC-L01, Peccel Technologies, Inc., Kawasaki, Japan). Spectral mismatch correction was applied by calibrating the AM 1.5 solar simulator (Peccel Technologies, Inc.) using a certified silicon (Newport Corporation, Irvine, CA, USA) reference cell (NREL). The simulator’s spectral output was compared to the standard AM 1.5 reference spectrum, and a correction factor was implemented to match the incident light intensity and spectrum. Light intensity (100 mW/cm^2^) was calibrated prior to each measurement session, and all *J–V* measurements were performed at an ambient temperature of 25 °C. Measurements were conducted on a masked active area of 0.25 cm^2^ (Figure S1). X-ray diffraction (XRD) analysis of f-TNAs was performed using a high-performance Rigaku diffractometer with Cu-Kα radiation (Rigaku Corporation, Tokyo, Japan). The dye loading concentration on f-TNAs was quantified via ultraviolet-visible (UV-Vis) spectroscopy using a NEOSYS-2000 spectrophotometer (SCINCO, Seoul, Republic of Korea). The calibration curve for N719 dye in 0.05 M NaOH (Sigma-Aldrich) used for quantifying dye loading is provided in Supporting Information (Figure S2). The method shows excellent linearity (*R^2^* = 0.998) and enables precise calculation of dye loading from measured absorbance values. Samples for dye loading concentration measurements were prepared by immersing the substrate in 0.05 M NaOH solution to desorb the dye. Electrochemical impedance spectroscopy (EIS) measurements were conducted using a Solartron 1287 potentiostat (Ametek, Farnborough, United Kingdom) coupled with a Solartron 1260 frequency response analyzer. The frequency range spanned from 10^−2^ Hz to 10 MHz, with an applied bias voltage set to the open-circuit voltage (*V_oc_*) and an AC amplitude of 10 mV. All impedance measurements were performed under AM 1.5 one-sun illumination conditions.

## 3. Results and Discussion

**Figure 1:**
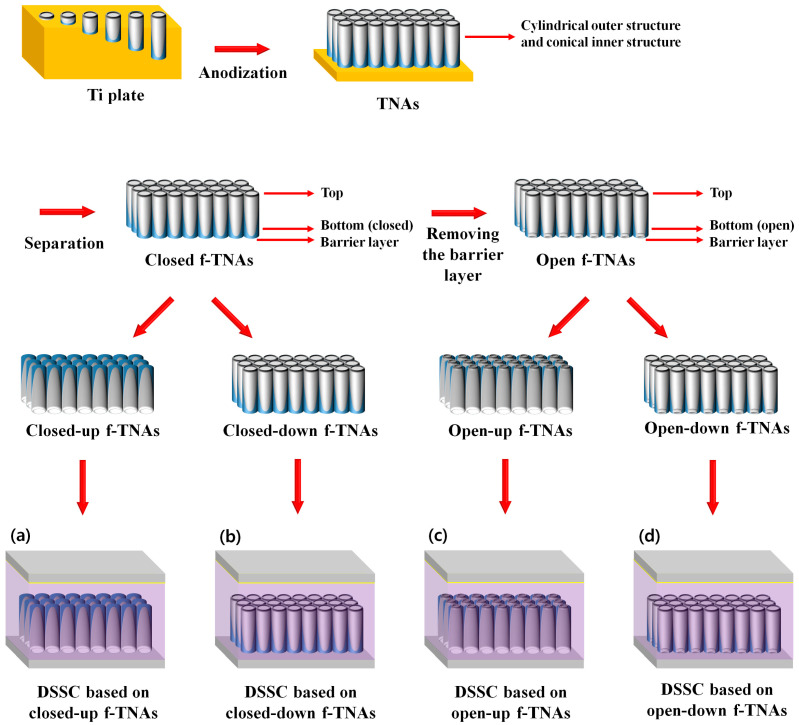
Overall scheme of DSSCs based on (**a**) closed-up, (**b**) closed-down, (**c**) open-up, and (**d**) open-down f-TNAs.

A schematic representation of dye-sensitized solar cells (DSSCs) incorporating freestanding TiO_2_ nanotube arrays (f-TNAs) is depicted in Figure 1. The TNAs were synthesized via anodization and subsequently detached from the Ti plate to yield f-TNAs. The f-TNAs exhibit a distinctive morphology characterized by a cylindrical outer structure and a conical inner structure, resulting from the interplay between field-assisted oxidation and dissolution processes. The formation mechanism of TNAs can be divided into two primary processes. The underlying mechanism for the formation of anodic TiO_2_ nanotube arrays is well established and can be attributed to the conventional field-assisted oxidation and dissolution model. Detailed equations and derivations related to this mechanism are provided in the Supporting Information for clarity [31,32].

DSSCs were fabricated using closed or open f-TNAs in four configurations: closed-up f-TNAs, closed-down f-TNAs, open-up f-TNAs, and open-down f-TNAs. DSSCs fabricated with closed f-TNAs, where the bottom is not open and is in a reversed structure on the fluorine-doped tin oxide (FTO) glass, are referred to as ‘DSSCs based on closed-up f-TNAs,’ as shown in Figure 1a. Similarly, DSSCs fabricated with closed f-TNAs in a normal structure on the FTO glass, where the bottom is not open, are referred to as ‘DSSCs based on closed-down f-TNAs,’ as shown in Figure 1b. DSSCs fabricated with open f-TNAs, where the bottom is open and in a reversed structure on the FTO glass, are referred to as ‘DSSCs based on open-up f-TNAs,’ as shown in Figure 1c. Similarly, DSSCs fabricated with open f-TNAs in a normal structure on the FTO glass, where the bottom is open, are referred to as ‘DSSCs based on open-down f-TNAs,’ as shown in Figure 1d.

Regarding the effects of the structural configuration of f-TNAs in DSSCs, the first consideration is dye adsorption. Dye adsorption is conducted after the annealing process to secure the f-TNAs on FTO glass. If dye adsorption is conducted before the annealing step, the dye decomposes during the annealing process. However, after the annealing process, the structural configuration of f-TNAs influences dye adsorption. The second effect concerns electrolyte diffusion during DSSC operation. For DSSCs to function under light exposure, a redox reaction takes place within the electrolyte, which depends on electrolyte diffusion, a process also affected by the structural configuration of f-TNAs. Accordingly, we prepared DSSCs based on four f-TNAs configurations: closed-up, closed-down, open-up, and open-down.

**Figure 2:**
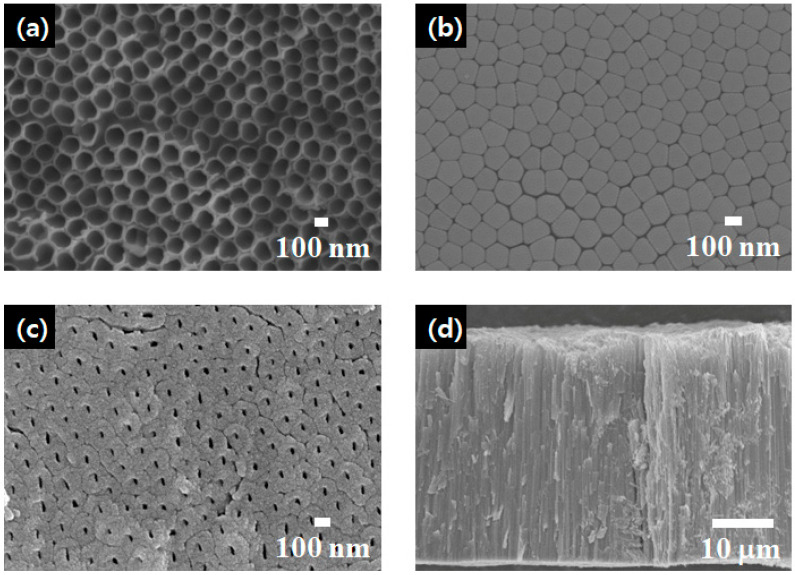
FE-SEM images of the top (**a**), bottom with barrier layer (**b**), bottom without barrier layer (**c**), and side view (**d**) of f-TNAs.

The morphology of the f-TNAs was characterized using field emission scanning electron microscopy (FE-SEM), as shown in Figure 2. The top of the f-TNAs has a pore size of 150 nm, as shown in Figure 2a. In contrast, the bottom, which includes a barrier layer, also has a thickness of 150 nm, as shown in Figure 2b. Notably, the barrier layer remains intact beneath the bottom of the f-TNAs after the anodization. After the ion milling, the barrier layer is removed and the bottom of f-TNAs is opened. The bottom without the barrier layer retains a size of 150 nm; however, the pore size in the bottom is 25 nm, as shown in Figure 2c. Since the outer diameter of f-TNAs is consistently 150 nm, the f-TNAs exhibit a cylindrical structure, as shown in Figure 2a,b. However, Figure 2c shows that the inner nanotube structure of the f-TNAs is conical, where the pore size at the top is significantly larger than at the bottom. Additionally, the wall thickness at the top (5 nm) is thinner compared to the 62 nm wall thickness at the bottom. The total length of the f-TNAs is 37 μm, as shown in Figure 2d. Overall, the external structure of the f-TNAs is cylindrical, while the internal structure is conical. The pore size at the top is approximately 150 nm, with a wall thickness of about 15 nm. At the bottom, the pore size is around 25 nm, and the wall thickness is approximately 70 nm. The total length of the f-TNAs is 37 μm. The thickness of the TiO_2_ compact layer is approximately 80 nm, while the TiO_2_ nanoparticle layer has a thickness of 3 μm, as determined after thermal treatment.

The crystallinity of TNAs is an important factor in preparing f-TNAs. After the 1st anodization of the Ti plate, an initial layer of amorphous TNAs forms on the Ti plate without crystallinity. In this state, the initial layer of amorphous TNAs is not only highly unstable in chemical reagents but also exhibits low electron transport in DSSCs [33]. However, following an annealing step, the initial layer of amorphous TNAs develops crystallinity, referred to as a first layer of crystalline TNAs, becoming more stable in chemical environments. To prepare f-TNAs, a 2nd anodization is required, which subsequently takes place in an electrolyte. In the 2nd anodization, the first layer of crystalline TNAs is more stable within the electrolyte. After the 2nd anodization, a second layer of amorphous TNAs forms at the base of the first layer of crystalline TNAs. As the second layer of amorphous TNAs is unstable in chemical reagents, it is removed using oxidizing agents. Once the second layer of amorphous TNAs is removed, the first layer of crystalline TNAs is detached from the Ti plate, resulting in f-TNAs with improved structural and electron properties suitable for application in DSSCs.

**Figure 3:**
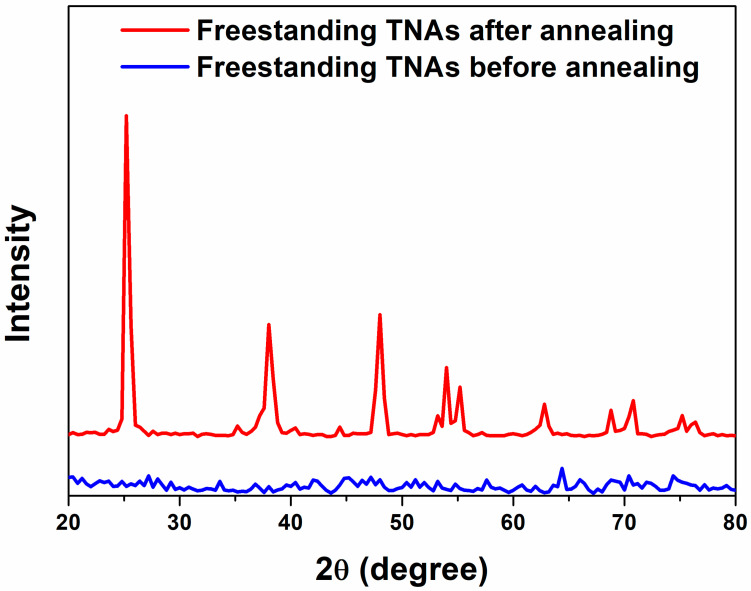
XRD patterns of f-TNAs before the annealing step (blue line) and after the annealing step (red line).

Figure 3 shows the crystallinity of f-TNAs with and without the annealing step, as characterized by X-ray diffraction (XRD). Before the annealing step, the f-TNAs exhibited amorphous structure, as represented by the blue line. After the annealing step, crystallinity significantly improves, as depicted by the red line. The crystal structure with the largest intensity peak is appeared in the (101) plane corresponding to 25°. The crystal planes of f-TNAs after annealing at (004), (200), (105), (211), (204), (116), (220), and (215) corresponding to 2θ angles 37.74°, 48.02°, 53.82°, 55.10°, 62.56°, 68.82°, 70.18°, and 75.18° confirm the anatase phase of f-TNAs, respectively [34]. After the barrier layer under the bottom layer of f-TNAs is removed by the ion milling, the crystallinity remains consistent with that of the f-TNAs that still have the barrier layer. This indicates that the removal of the barrier layer does not affect the overall crystallinity of the f-TNAs.

**Figure 4:**
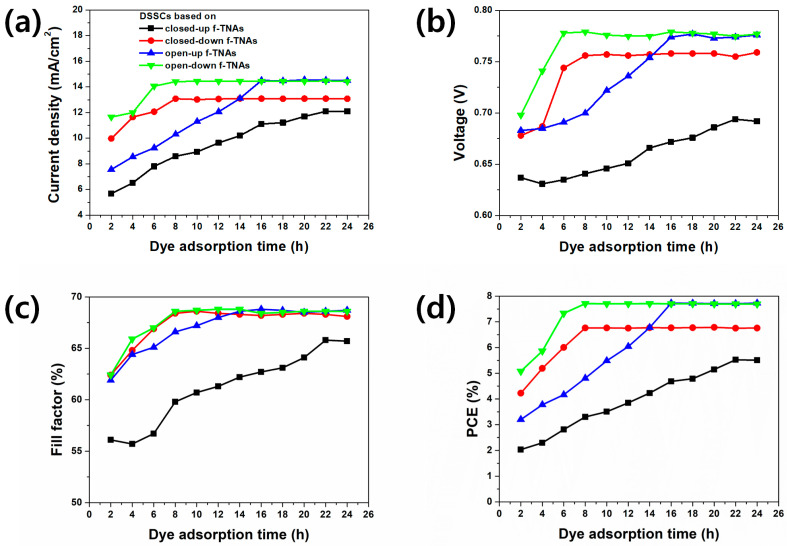
I-V data of DSSCs based on closed-up, closed-down, open-up, and open-down f-TNAs. (**a**) represents *J_sc_*, (**b**) represents *V_oc_*, (**c**) represents FF, and (**d**) represents PCE. The black line indicates DSSCs based on closed-up f-TNAs, the red line indicates DSSCs based on closed-down f-TNAs, the blue line indicates DSSCs based on open-up f-TNAs, and the green line indicates DSSCs based on open-down f-TNAs.

The parameters of DSSCs based on closed-up, closed-down, open-up, and open-down f-TNAs, including current density (*J_sc_*), open-circuit voltage (*V_oc_*), fill factor (FF), and power conversion efficiency (PCE), are summarized in Tables S1–S4. The best PCE values for DSSCs using these different configurations of f-TNAs are presented in Figure 4 and summarized in Table 1. The dye adsorption time ranged from 2 to 24 h. Ten samples were fabricated for each configuration, and the standard deviation of the PCE for each sample is shown in Table 1. Power conversion efficiency (PCE, %) of DSSCs as a function of dye adsorption time for closed-up, open-up, closed-down, and open-down freestanding TiO_2_ nanotube arrays (f-TNAs) is shown in Figure S3. Error bars represent standard deviation from replicate measurements.

After 2 h of dye adsorption time, the *J_sc_*, *V_oc_*, FF, and PCE had the lowest values for all DSSCs. The PCE of DSSCs based on closed-down and open-down f-TNAs showed their best performance after 8 h of dye adsorption, reaching PCE of 6.78% and 7.71%, respectively. The DSSCs based on open-up f-TNAs showed the maximum PCE of 7.73% after 16 h of dye adsorption, whereas the DSSC based on closed-up f-TNAs achieved its best PCE of 5.52% after 22 h of dye adsorption time. Overall, the PCE increased with longer dye adsorption times. DSSCs based on closed-down, closed-up, open-down, and open-up f-TNAs demonstrated the highest PCEs after sufficient dye adsorption time. The highest PCE of DSSCs based on closed-down f-TNAs requires a dye adsorption time of 8 h, while for DSSCs based on closed-up f-TNAs require 22 h. In this case, the PCE of DSSCs based on closed-down f-TNAs is higher than that based on closed-up f-TNAs.

The PCE of DSSCs is significantly influenced by the structural configuration of f-TNAs. DSSCs based on closed-up f-TNAs exhibit diminished PCE, primarily due to constraints in dye adsorption and electrolyte diffusion during operation. Similarly, DSSCs based on closed-down f-TNAs yield lower PCE compared to DSSCs based on open-up or open-down f-TNAs, potentially attributable to the presence of a barrier layer under the bottom of the f-TNAs base. Interestingly, DSSCs based on open-up and open-down f-TNAs demonstrate comparable PCE values after sufficient dye adsorption periods. While DSSCs based on open-down f-TNAs initially show higher PCE than open-up configurations at 8 h, both structures converge to identical PCE values at 16 h. Moreover, the maximum efficiency of ~7.73% achieved in the open configurations lies within the upper range of typical TiO_2_ nanotube-based DSSCs (generally reported around 6–8%), highlighting that our devices reach competitive performance levels solely through structural optimization without the need for additional dopants or co-sensitizers. The relationship between f-TNAs structural configurations and dye adsorption is further corroborated by time-dependent dye loading measurements.

To evaluate the long-term operational stability of the DSSCs, each device was subjected to continuous one-sun light-soaking tests according to the ISOS-L2 [35] protocol. The PCE values were measured at several time intervals over 120 h. As summarized in Table S5, all devices exhibited gradual decreases in PCE with extended irradiation, but maintained high efficiency retention rates of 86–88% after 120 h. These results demonstrate the robust long-term stability and durability of the barrier-layer-free f-TNA-based DSSCs under prolonged operating conditions. Barrier-layer-free TiO_2_ nanotube DSSCs exhibit excellent short-term stability with over 90% PCE retention after 72–120 h of light soaking when properly fabricated and sealed, but their long-term durability may still be affected by electrochemical or environmental degradation if structural or sealing defects are present.

Details of the EQE spectra and integrated *J_sc_* values for all DSSC configurations are provided in the Supporting Information (Figure S4). The integrated *J_sc_* derived from the EQE spectra closely matches the *J_sc_* obtained from *J–V* measurements, confirming the reliability of the photocurrent response and the effect of nanotube structure on spectral performance.

**Figure 5:**
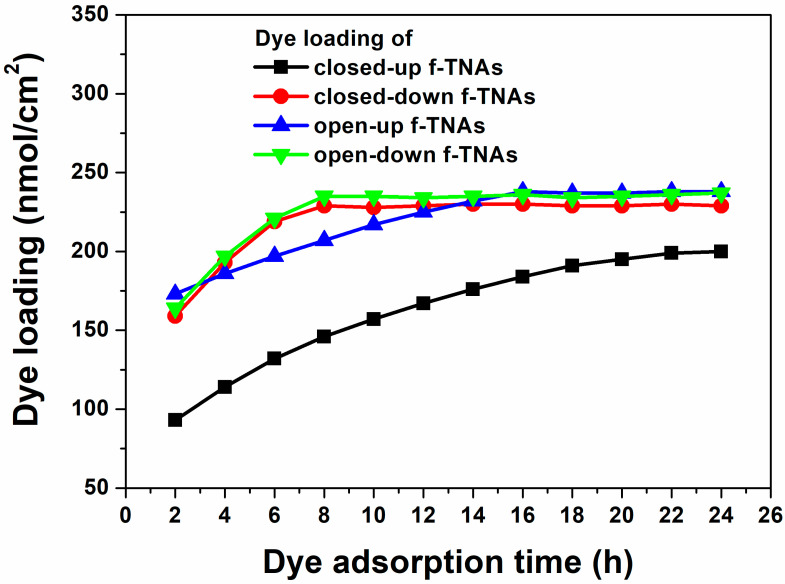
Dye loading on closed-up (black line), closed-down (blue line), open-up (red line), and open-down (green line) f-TNAs as a function of dye adsorption time.

Dye loading was measured at various intervals to investigate how dye adsorption time affects the performance of DSSCs based on closed-up, closed-down, open-up, and open-down f-TNAs, as shown in Figure 5 and summarized in Table S6. The amount of dye is related to *J_sc_*, *V_oc_*, FF, and PCE in DSSCs.

The dye loading of closed-up f-TNAs increases from 93 nmol/cm^2^ to 199 nmol/cm^2^ up to 22 h. At the same time, the *J_sc_*, *V_oc_*, FF, and PCE of DSSCs based on closed-up f-TNAs also increase from 5.68 mA/cm^2^ to 12.10 mA/cm^2^, 0.64 V to 0.694 V, 56.1% to 65.8%, and 2.03% to 5.52%, respectively. Considering the dye adsorption rate of about 4.818 nmol/h, the dye adsorption is hindered by closed-up f-TNAs, preventing sufficient dye adsorption. On closed-up TNAs, the dye solution has difficulty penetrating the inner surface of f-TNAs. As a result, the dyes are first adsorbed on the outer surface of f-TNAs and then slowly adsorbed on the inner surface of f-TNAs during the long dye adsorption time. Nevertheless, the best PCE (5.52%) of DSSCs based on closed-up f-TNAs is still lower than that of any other DSSCs with f-TNAs. This highlights the control of dye adsorption imposed by the structural design of closed-up f-TNAs in facilitating effective dye loading and, consequently, optimal PCE.

The dye loading of closed-down f-TNAs increases from 159 nmol/cm^2^ to 229 nmol/cm^2^ up to 8 h. Accordingly, *J_sc_*, *V_oc_*, FF, and PCE of DSSCs based on closed-down f-TNAs also increase from 9.99 mA/cm^2^ to 13.08 mA/cm^2^, 0.68 V to 0.76 V, 62.4% to 68.4%, and 4.22% to 6.76%, respectively. The best PCE of DSSCs based on closed-down f-TNAs is 6.78%, with *J_sc_*, *V_oc_*, and FF of 13.09 mA/cm^2^, 0.76 V, and 68.4%, respectively. The dye adsorption rate of closed-down f-TNAs is about 8.75 nmol/h, which is faster than that of closed-up f-TNAs. In this case, the pore size at the top of f-TNAs is 150 nm, which means the dyes are smoothly permeated into the channels of f-TNAs and easily adsorbed on the inner and outer surfaces of f-TNAs. After 8 h, the dye loading does not increase further, and the PCE of DSSCs remains similar to that of other DSSCs.

For open-up f-TNAs, the dye loading increases from 173 nmol/cm^2^ to 238 nmol/cm^2^ up to 16 h. The *J_sc_*, *V_oc_*, FF, and PCE of DSSCs based on open-up f-TNAs also increase from 7.57 mA/cm^2^ to 14.51 mA/cm^2^, 0.68 V to 0.77 V, 61.9% to 68.8%, and 3.20% to 7.73%, respectively. The best PCE of DSSCs based on open-up f-TNAs is 7.73%, with *J_sc_*, *V_oc_*, and FF of 14.50 mA/cm^2^, 0.78 V, and 68.7%, respectively. The dye adsorption rate of open-up f-TNAs is 4.06 nmol/h, which is lower than that of closed-down f-TNAs despite the bottom layer of f-TNAs being removed and fully opened. However, after 2 h, the dye loading is considerably higher than in any other f-TNAs. This suggests that while the dye adsorption rate is moderate, the open-up structure effectively enhances initial dye absorption.

After anodization, the shape of TNAs has a cylindrical structure, meaning both the top and bottom have the same size. However, the pore size of the top TNAs is 150 nm, while the pore size of the bottom TNAs is 25 nm. In other words, the wall thickness of the top f-TNAs (about 5 nm) is thin, and the wall thickness of the bottom f-TNAs (62 nm) is thick, as shown in Figure 2a,c. In closed-down f-TNAs, the dye solution passes through the pores of bottom f-TNAs, and the dye is adsorbed on the surface of the bottom of f-TNAs. Due to the substantial wall thickness at the bottom, the initial dye loading amount is much higher compared to other f-TNA configurations after 2 h. However, before 16 h, the amount of dye loading increases slowly due to the thin wall thickness of f-TNAs. After 16 h, no further increase in dye loading is observed, and the PCE of the DSSCs stabilizes, remaining similar to that of DSSCs using other types of f-TNAs.

The dye loading of open-down f-TNAs increases from 164 nmol/cm^2^ to 235 nmol/cm^2^ up to 8 h. The *J_sc_*, *V_oc_*, FF, and PCE of DSSCs based on open-down f-TNAs also increase from 11.66 mA/cm^2^ to 14.42 mA/cm^2^, from 0.70 V to 0.78 V, from 62.4% to 68.6%, and from 5.08% to 7.71%, respectively. The best PCE of DSSCs based on open-down f-TNAs is 7.71%, with *J_sc_*, *V_oc_*, and FF of 14.42 mA/cm^2^, 0.78 V, and 68.6%, respectively. The dye adsorption rate of open-down f-TNAs is about 8.87 nmol/h, which is higher than that of the closed-up f-TNAs. In this case, the pore size at the top of f-TNAs is 150 nm, which means the dyes are smoothly permeated into the channels of f-TNAs and easily adsorbed on the inner and outer surfaces of f-TNAs, similar to the closed-up f-TNAs. After 8 h, the dye loading does not increase further, and the PCE of DSSCs remains similar to that of other DSSCs.

**Figure 6:**
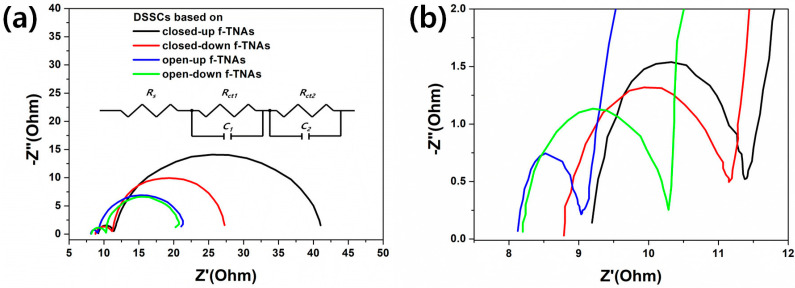
Impedance of DSSCs based on closed-up (black line), closed-down (red line), open-up (blue line), and open-down (green line) f-TNAs. (**a**) shows the total impedance of DSSCs and (**b**) is an enlargement of the small circle in the impedance plot.

The PCE of DSSCs based on closed-up, closed-down, open-up, and open-down f-TNAs is influenced by the dye loading, which directly affects the *J_sc_*. However, the electron transport or transfer could not be explained solely by dye loading. Instead, the electron transport or transfer is dependent on the resistance in the structure of DSSCs. Figure 6 shows the Nyquist plots of the DSSCs based on different f-TNAs, with the corresponding equivalent circuit illustrated in the inset. In this circuit, Rs represents the series resistance originating from the conductive substrate, wiring, and electrolyte, and is typically extracted from the high-frequency intercept of the plot. *R*_*ct*1_ and *C*_1_ model the charge-transfer resistance and double-layer capacitance, respectively, at the counter electrode (Pt/electrolyte) interface, corresponding to the first semicircle in the Nyquist plot. *R*_*ct*2_ and *C*_2_ represent the charge-transfer resistance and interfacial capacitance at the photoanode/electrolyte (TiO_2_ NTs/electrolyte) interface and are responsible for the second semicircle. This two-RC circuit effectively separates the interfacial kinetics of the counter electrode and photoanode, consistent with the two distinct semicircles observed experimentally. The detailed fitting parameters, including *R_s_*, *R*_*ct*1_, *C*_1_, *R*_*ct*2_, and *C*_2_, as well as the goodness-of-fit indicators (*χ*^2^, *R*^2^), are summarized in Table 2. Each value is reported with its associated fitting uncertainty (± standard error) as estimated from the fitting algorithm. The very low *χ*^2^ values and high *R*^2^ values confirm the excellent quality of the model fit. Warburg-type (diffusion) elements were not included, as our spectra are dominated by charge-transfer processes under these conditions. This equivalent circuit approach is in line with standard practice in DSSC EIS analysis and allows us to quantitatively compare the electrochemical properties of the different f-TNA architectures [36,37]. The DSSC based on closed-up f-TNAs shows high *R_s_* (9.04 Ω), *R*_*ct*1_ (2.58 Ω), and *R*_*ct*2_ (28.72 Ω), respectively. The f-TNAs in DSSCs have a reverse structure, where the top is in contact with the FTO glass, and the bottom is in contact with the electrolyte. The electrons generated on dyes circulate through f-TNAs, FTO electrode, Pt electrode, electrolyte, and back to dyes [38]. During the redox process in the electrolyte, the dyes are reduced by electrons. However, in DSSCs based on closed-up f-TNAs, the electrolyte is not able to easily penetrate the inner dyes of f-TNAs due to the lack of pores on the bottom of closed-up f-TNAs. Consequently, the resistances *R_s_*, *R*_*ct*1_, and *R*_*ct*2_ have higher values compared to other DSSCs with f-TNAs. The DSSCs based on closed-down f-TNAs show lower *R_s_* (8.69 Ω), *R*_*ct*1_ (2.38 Ω), and *R*_*ct*2_ (17.32 Ω), respectively. The f-TNAs have a normal structure, which means the bottom is in contact with the FTO glass and the top is in contact with the electrolyte. The electrolyte can easily penetrate to the inner dyes of f-TNAs through the top pores of f-TNAs, and the dyes are easily reduced by electrons during the redox process. Consequently, the resistance *R*_*ct*2_ of closed-down f-TNAs is decreased compared to the *R*_*ct*2_ of closed-up f-TNAs. The DSSCs based on open-up f-TNAs show *R_s_* (8.00 Ω), *R*_*ct*1_ (1.12 Ω), and *R*_*ct*2_ (12.88 Ω), respectively. The f-TNAs have a reverse structure, which means the top is in contact with the FTO glass, and the bottom is in contact with the electrolyte. Compared to the closed-up f-TNAs, the difference is that the bottom is open with a pore size of 25 nm. The electrolyte can easily penetrate the inner dyes of f-TNAs through the bottom pores of f-TNAs, and the dyes are reduced by electrons during the redox process. Consequently, the resistance *R*_*ct*2_ of open-up f-TNAs is lower compared to the *R*_*ct*2_ of closed-up or closed-down f-TNAs. The DSSCs based on open-down f-TNAs show *R_s_* (8.11 Ω), *R*_*ct*1_ (2.06 Ω), and *R*_*ct*2_ (11.19 Ω). The f-TNAs have a normal structure, which means the bottom is in contact with the FTO glass, and the top is in contact with the electrolyte. The electrolyte can easily penetrate to the inner dyes of f-TNAs through the top pores of f-TNAs, and the dyes are easily reduced by electrons during the redox process. Consequently, the resistance *R*_*ct*2_ of open-down f-TNAs is lower compared to the *R*_*ct*2_ of closed-up or closed-down f-TNAs. The structural orientation facilitates better electrolyte infiltration and more efficient electron transport, thereby reducing resistances and enhancing the DSSC performance.

Compared to the DSSCs based on closed-down or open-down f-TNAs, the dye adsorption time is 8 h, and the best PCE of DSSCs based on closed-down or open-down f-TNAs is 6.78% or 7.71%, despite the dye loading being 7 nmol/cm^2^. The difference between these two configurations is the presence of a barrier layer beneath the bottom of the closed-down f-TNAs. In this case, as the barrier layer significantly impedes the electron transfer from f-TNAs to FTO glass, the *R*_*ct*1_ (2.06 Ω) of DSSCs based on open-down f-TNAs is lower than that (2.38 Ω) of DSSCs based on closed-down f-TNAs.

Compared to the DSSCs based on open-up or open-down f-TNAs, both configurations achieve their optimal power conversion efficiency (PCE) and dye loading after sufficient dye adsorption times: 7.73% with 238 nmol/cm^2^ for open-up f-TNAs after 16 h and 7.71% with 235 nmol/cm^2^ for open-down f-TNAs after 8 h. The resistance of DSSCs based on open-down f-TNAs shows a slight difference compared to DSSCs based on open-up f-TNAs. Especially, the *R*_*ct*1_ (2.06 Ω) of DSSCs based on open-down f-TNAs is higher than that (1.12 Ω) of DSSCs based on open-up f-TNAs. In contrast, the *R*_*ct*2_ (11.19 Ω) of DSSCs based on open-down f-TNAs is lower than that (12.88 Ω) of DSSCs based on open-up f-TNAs. The f-TNAs have a cylindrical structure featuring a conical inner design. The top wall is thin (5 nm), which leads to a large pore size of 150 nm. In contrast, the bottom wall is thicker (approximately 62 nm), resulting in a smaller pore size of 25 nm. Consequently, the structural configurations of the f-TNAs influence the parameters of DSSCs, and these parameters are verified through resistance measurements. *R*_*ct*1_ is the resistance at the FTO/f-TNAs/dye interface. Low resistance indicates low recombination because a thin wall is advantageous for charge carrier transport, resulting in reduced recombination, better charge separation, and faster electron transport. Consequently, the DSSCs based on open-up f-TNAs are effective for *R*_*ct*1_. *R*_*ct*2_ is the resistance at the TNAs/dye/electrolyte interface. To reduce the resistance of *R*_*ct*2_, the interface between the dye and electrolyte should not be disturbed, as the large pore size is suitable for electrolyte diffusion. Consequently, the open-down f-TNAs are beneficial for *R*_*ct*2_.

### 3.1. Structural Configurations and Performance Characteristics of f-TNAs

This study investigates the impact of various structural configurations of f-TNAs on the performance in DSSCs. Four distinct configurations are examined: closed-up, closed-down, open-up, and open-down f-TNAs.

#### 3.1.1. Closed-Up f-TNAs

**Figure 7:**
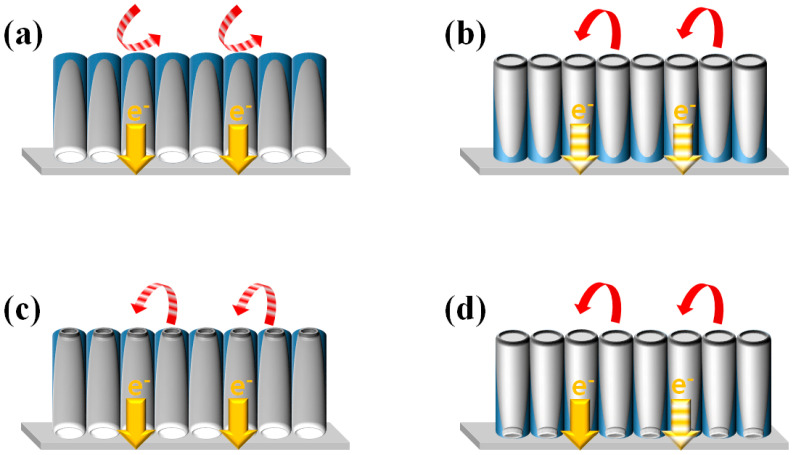
The structural configuration effects of f-TNAs on DSSCs. (**a**) closed-up, (**b**) closed-down, (**c**) open-up, and (**d**) open-down f-TNAs on DSSCs.

In the closed-up configuration, the bottom of the f-TNAs forms the upper part, while the top constitutes the lower part. This structure significantly impedes dye adsorption and electrolyte diffusion due to the closed bottom, as illustrated in Figure 7a. Consequently, electron generation is limited to the outer surface of the f-TNAs, where dye molecules are adsorbed. The redox coupling reaction is similarly confined to the outer surface. Despite these limitations, the thin walls of the f-TNAs facilitate efficient electron transfer to the electrode.

#### 3.1.2. Closed-Down f-TNAs

The closed-down configuration presents the top of the f-TNAs as the upper part and the bottom as the lower part. This arrangement, depicted in Figure 7b, allows for enhanced dye adsorption and electrolyte diffusion through the large top pore. As a result, electron generation occurs on both the inner and outer surfaces of the f-TNAs. The redox coupling reaction also takes place on both surfaces. However, the presence of a barrier layer at the bottom of the f-TNAs restricts electron transfer to the electrode.

#### 3.1.3. Open-Up f-TNAs

In the open-up configuration, the bottom of the f-TNAs forms the upper part, while the top constitutes the lower part. As shown in Figure 7c, this structure allows for slow penetration of dye molecules and electrolyte through the small bottom pore. Electron generation occurs on both inner and outer surfaces, but only after an extended dye adsorption period. The redox coupling reaction is impeded by the small bottom pore. Nevertheless, the thin walls of the f-TNAs ensure efficient electron transfer to the electrode.

#### 3.1.4. Open-Down f-TNAs

The open-down configuration presents the top of the f-TNAs as the upper part and the bottom as the lower part. As illustrated in Figure 7d, this structure facilitates easy penetration of dye molecules and electrolyte through the large top pore. Consequently, electron generation and redox coupling reactions occur on both inner and outer surfaces of the f-TNAs. However, the thick walls of the f-TNAs in this configuration constrain electron transfer to the electrode.

These findings elucidate the complex interplay between structural configurations of f-TNAs and their performance characteristics in DSSCs. The study highlights the importance of optimizing the f-TNA structure to balance dye adsorption, electrolyte diffusion, and electron transfer processes for enhanced DSSC efficiency.

## 4. Conclusions

In conclusion, this systematic investigation reveals that both the orientation and openness of freestanding TiO_2_ nanotube arrays are pivotal for optimizing dye adsorption efficiency and charge transport, therein enhancing DSSC device performance. Our results establish clear mechanistic links between nanotube morphology, dye loading, and interfacial charge transfer processes, thereby providing a valuable framework for rational electrode design. Importantly, the key design principle uncovered in this work is that removing the barrier layer and optimizing array orientation to employ f-TNAs with an open-bottom structure (open-up configuration) substantially maximizes dye loading, facilitates electrolyte penetration, and minimizes recombination—all crucial for achieving high power conversion efficiency. We therefore recommend future high-performance DSSC photoanodes adopt such open-bottom, optimally engineered architectures. Nevertheless, the present study is limited by the absence of direct diffusion modeling and scale-up validation, and further quantitative analyses are needed to fully elucidate structural impacts and reproducibility for larger-area devices. We anticipate that these insights will accelerate the development of next-generation DSSC materials with enhanced efficiency and reliability.

## Figures and Tables

**Table 1:** Best parameters of DSSCs based on closed-up, closed-down, open-up, and open-down f-TNAs.

DSSCs Based on	*J_sc_* (mA/cm^2^)	*V_oc_* (V)	FF (%)	PCE (%)
closed-up f-TNAs	12.10	0.70	65.8	5.52 ± 0.22
closed-down f-TNAs	13.09	0.76	68.4	6.78 ± 0.28
open-up f-TNAs	14.50	0.78	68.7	7.73 ± 0.15
open-down f-TNAs	14.42	0.78	68.6	7.71 ± 0.36

**Table 2:** Impedance of DSSCs based on closed-up, closed-down, open-up, and open-down f-TNAs.

DSSCs Based on	*R_s_* (Ω)	*R*_*ct*1_ (Ω)	*C*_1_ (μF)	*R*_*ct*2_ (Ω)	*C*_2_ (μF)	*χ* ^2^	*R* ^2^
closed-up f-TNAs	9.04 ± 0.15	2.58 ± 0.22	4.42 ± 0.35	28.72 ± 1.31	6.68 ± 0.45	0.0012	0.997
closed-down f-TNAs	8.69 ± 0.13	2.38 ± 0.15	3.98 ± 0.28	17.32 ± 1.04	5.52 ± 0.39	0.0010	0.995
open-up f-TNAs	8.00 ± 0.10	1.12 ± 0.10	1.74 ± 0.16	12.88 ±0.63	4.03 ± 0.32	0.0009	0.998
open-down f-TNAs	8.11 ± 0.09	2.06 ± 0.17	2.93 ± 0.23	11.19 ± 0.59	3.47 ± 0.29	0.0011	0.998

## Data Availability

The original contributions presented in this study are included in the article. Further inquiries can be directed to the corresponding authors.

## Supplementary Materials

The following supporting information can be downloaded at: https://www.mdpi.com/article/10.3390/ma18225101/s1. Figure S1: (a) Optical image of a DSSC device with an active area of 0.25 cm^2^, (b) a DSSC device with an active area of 4 cm^2^, (c) the 0.25 cm^2^ aperture mask. Figure S2: Calibration curve of N719 dye absorbance at 540 nm in 0.05 M NaOH. Table S1: DSSCs based on closed-up f-TNAs by dye adsorption time. Table S2: DSSCs based on closed-down f-TNAs by dye adsorption time. Table S3: DSSCs based on open-up f-TNAs by dye adsorption time. Table S4: DSSCs based on open-down f-TNAs by adsorption time. Figure S3: Power conversion efficiency (PCE, %) of DSSCs as a function of dye adsorption time for (a) closed-up, (b) open-up, (c) closed-down, and (d) open-down freestanding TiO_2_ nanotube arrays (f-TNAs). Error bars represent standard deviation from replicate measurements. Table S5: Time-dependent PCE and retention of DSSCs during extended one-sun light-soaking. Figure S4: EQE spectra and integrated *J_sc_* of DSSCs based on closed-up (black), closed-down (red), open-up (blue), and open-down (green) f-TNAs. Table S6: Amount of dye loading on closed-up, closed-down, open-up, and open-down f-TNAs by dye adsorption time.
