# Supplementary material for: Structural Configuration Effects of Freestanding TiO2 Nanotube Arrays on Power Conversion Efficiency in Dye-Sensitized Solar Cells
Source: Materials (Basel). 2025 Nov 10;18(22):5101. doi: 10.3390/ma18225101 (PMC12654371; doi:10.3390/ma18225101)
Supplement: Supplementary file 1 [file materials-18-05101-s001.zip › materials-3938607-supplementary.pdf]

Supporting information

Structural Configuration Effects of Freestanding TiO_2 Nanotube Arrays on Power Conversion Efficiency in Dye-Sensitized Solar Cells

Gangasagar Sharma Gaudel [1,†], Seung-Ju Yu [1,†], Hwa-Young Yang [2,†], Ye-Chong Moon [1], Sang Hoon Kim [3], Sang-Ho Park [4,*], Bong-Hyun Jun [5,*], Young Jun Kim [6,*], Won-Yeop Rho [1,7,*]

[1] Graduate School of Integrated Energy-AI, Jeonbuk National University, Jeonju-si 54896, Republic of Korea; gangasagarsg@gmail.com (G.S.G.); ysj__0708@naver.com (S.-J.Y.); catalystm47@gmail.com (Y.-C.M.);
[2] School of Energy and Chemical Engineering, Ulsan National Institute of Science and Technology (UNIST), Ulsan 44919, Republic of Korea; hyang@unist.ac.kr
[3] JCHI GLOBAL Co., Ltd., Insta I - 506ho, Incheon 22004, Republic of Korea; bndshkim@gmail.com
[4] School of Chemical Engineering, Jeonbuk National University, Jeonju-si 54896, Republic of Korea
[5] Department of Bioscience and Biotechnology, Konkuk University, Seoul 05029, Republic of Korea
[6] Environmental Safety Group, Korea Institute of Science and Technology Europe (KIST-europe), 66123 Saarbrücken, Germany
[7] School of International Engineering and Science, Jeonbuk National University, Jeonju-si 54896, Republic of Korea
* Correspondence: shpark92@jbnu.ac.kr (S.-H.P.); bjun@konkuk.ac.kr (B.-H.J.); youngjunkim@kist-europe.de (Y.J.K.); rho7272@jbnu.ac.kr (W.-Y.R.)
† These authors contributed equally to this work.

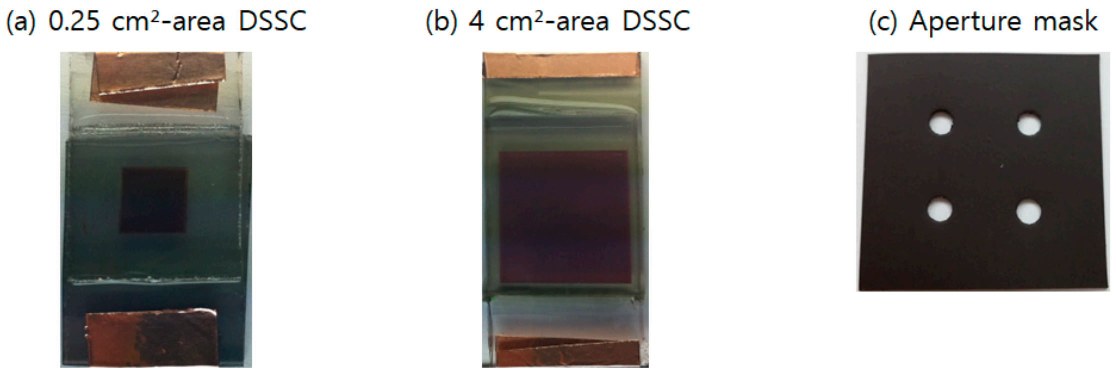

Figure S1. (**a**) Optical image of a DSSC device with an active area of 0.25 cm², (**b**) a DSSC device with an active area of 4 cm², (**c**) the 0.25 cm² aperture mask.

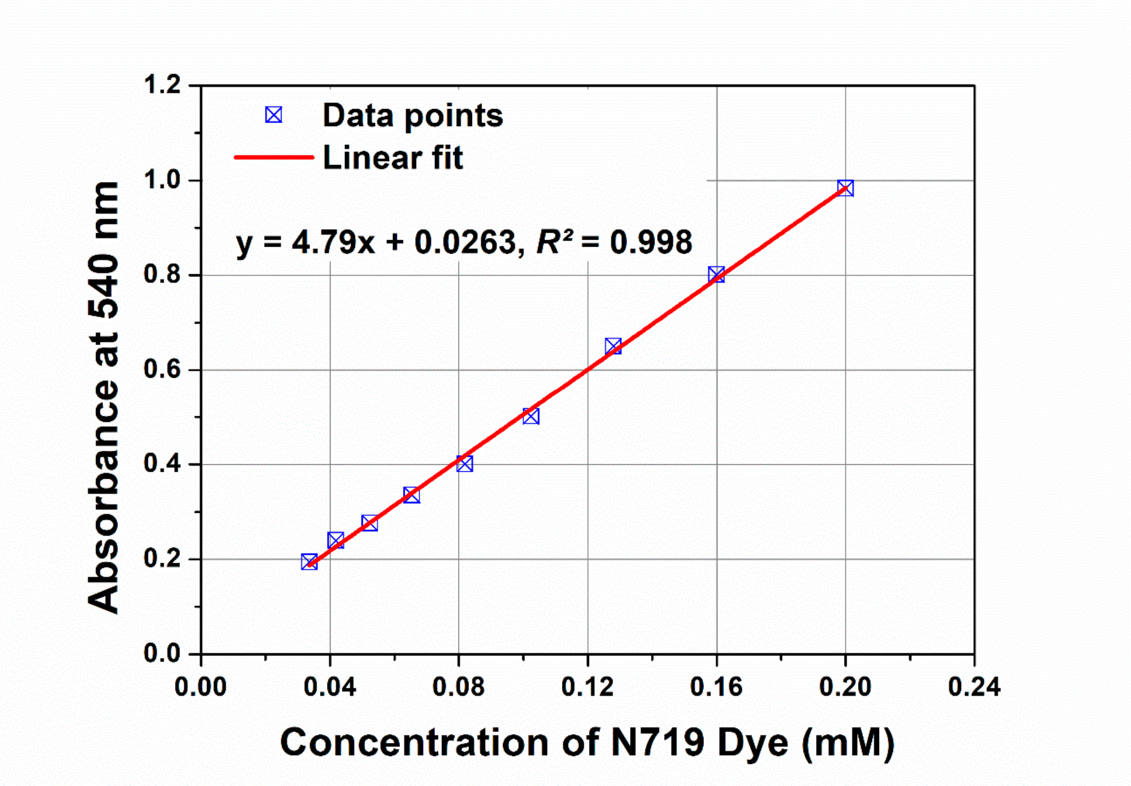

Figure S2. Calibration curve of N719 dye absorbance at 540 nm in 0.05 M NaOH.

Figure S2 shows the calibration curve for N719 dye absorbance at 540 nm in 0.05 M NaOH. Standard solutions of N719 dye were prepared at various concentrations and measured using a 1 cm path length quartz cuvette. The calibration curve demonstrates excellent linearity (R^2 = 0.998) within the range tested (0.0336–0.2 mM), confirming the reliability of the method for quantitative analysis of desorbed dye. The extinction coefficient (molar absorptivity) was determined to be 4.79 $mM^{-1}\cdot cm^{-1}$ from the slope of the linear fit. All measurements were replicated at least three times to ensure reproducibility, and the average values with standard deviation are reported. This calibration allows accurate calculation of dye loading from UV-Vis absorbance measurements following desorption in 0.05 M NaOH.

Field-Assisted Oxidation

During field-assisted oxidation, Ti undergoes transformation into TiO_2 at the anode through the following electrochemical reactions:

$$Ti \rightarrow Ti^{4+} + 4e^{-} \tag{1}$$

$$Ti^{4+} + 4OH^{-} \rightarrow Ti(OH)_4 \tag{2}$$

$$Ti^{4+} + 2O^{2-} \rightarrow TiO_2 \tag{3}$$

$$Ti(OH)_4 \rightarrow TiO_2 + 2H_2O \tag{4}$$

Concurrently, at the cathode:

$$4H^{+} + 4e^{-} \rightarrow 2H_2 \tag{5}$$

The overall reaction can be summarized as:

$$Ti + 2H_2O \rightarrow TiO_2 + 2H_2 \tag{6}$$

This process initiates the formation of a cylindrical TiO_2 film on the Ti plate surface, although the nanotube structure is not yet established.

Field-Assisted Dissolution

In the field-assisted dissolution phase, TiO_2 undergoes conversion to TiF_6^{2-} through the following reactions:

$$TiO_2 + 6F^{-} + 4H^{+} \rightarrow TiF_6^{2-} + H_2O \tag{7}$$

$$Ti(OH)_4 + 6F^{-} \rightarrow TiF_6^{2-} + 4OH^{-} \tag{8}$$

$$Ti^{4+} + 6F^{-} \rightarrow TiF_6^{2-} \tag{9}$$

The resulting TiF_6^{2-} species dissolve into the aqueous medium, facilitating the formation of the conical inner structure of the TiO_2 nanotubes. The dynamic equilibrium between field-assisted oxidation and dissolution processes gives rise to the unique cylindrical outer and conical inner structure of the TNAs on the titanium substrate.

Following separation from the Ti plate, the TNAs are designated as f-TNAs. The f-TNAs initially possess a barrier layer at their base. To enhance the functionality, ion milling is employed to remove the barrier layer, creating an open bottom structure. The post-processing step yields two distinct configurations:

1. Closed f-TNAs: Structures that retain the barrier layer, maintaining a closed bottom.
2. Open f-TNAs: Structures that have undergone ion milling, resulting in an open bottom configuration.

This modification significantly influences the properties and potential applications of the f-TNAs in DSSCs, allowing for improved electrolyte penetration and charge transport.

Although the f-TNAs have a barrier layer under the bottom, this layer is removed by an ion milling to open the bottom. Without the ion milling, the bottom remains closed, which is referred to as closed f-TNAs; with the ion milling, the bottom is open, which is referred to as open f-TNAs.

Table S1. DSSCs based on closed-up f-TNAs by dye adsorption time.

**Dye adsorption time (h)**	**J_{sc} (mA/cm²)**	**V_{oc} (V)**	**FF (%)**	**PCE (%)**
2	5.69	0.64	56.1	2.03
4	6.53	0.63	55.7	2.29
6	7.81	0.63	56.7	2.81
8	8.61	0.64	59.8	3.30
10	8.94	0.65	60.7	3.51
12	9.65	0.65	61.3	3.85
14	10.21	0.67	62.2	4.23
16	11.12	0.67	62.7	4.68
18	11.22	0.68	63.1	4.79
20	11.70	0.69	64.1	5.15
22	12.10	0.69	65.8	5.52
24	12.11	0.69	65.7	5.51

Table S2. DSSCs based on closed-down f-TNAs by dye adsorption time.

**Dye adsorption time (h)**	**J_{sc} (mA/cm²)**	**V_{oc} (V)**	**FF (%)**	**PCE (%)**
2	9.99	0.68	62.4	4.22
4	11.65	0.69	64.8	5.19
6	12.07	0.74	66.9	6.01
8	13.08	0.76	68.4	6.76
10	13.02	0.76	68.6	6.76
12	13.06	0.76	68.4	6.75
14	13.10	0.76	68.3	6.77
16	13.09	0.76	68.2	6.77
18	13.08	0.76	68.3	6.77
20	13.09	0.76	68.4	6.78
22	13.09	0.76	68.3	6.75
24	13.08	0.76	68.1	6.76

Table S3. DSSCs based on open-up f-TNAs by dye adsorption time.

Dye adsorption time (h)	J_{sc} (mA/cm²)	V_{oc} (V)	FF (%)	PCE (%)
2	7.57	0.68	61.9	3.20
4	8.56	0.68	64.4	3.78
6	9.25	0.69	65.1	4.16
8	10.31	0.70	66.6	4.81
10	11.31	0.72	67.2	5.49
12	12.07	0.74	68.0	6.04
14	13.11	0.75	68.6	6.78
16	14.51	0.77	68.8	7.73
18	14.47	0.78	68.7	7.72
20	14.56	0.77	68.5	7.71
22	14.52	0.77	68.6	7.71
24	14.50	0.78	68.7	7.73

Table S4. DSSCs based on open-down f-TNAs by adsorption time.

Dye adsorption time (h)	J_{sc} (mA/cm^2)	V_{oc} (V)	FF (%)	PCE (%)
2	11.66	0.70	62.4	5.08
4	12.01	0.74	65.9	5.86
6	14.06	0.78	67.0	7.33
8	14.42	0.78	68.6	7.71
10	14.45	0.78	68.7	7.70
12	14.45	0.77	68.8	7.70
14	14.46	0.77	68.8	7.71
16	14.45	0.78	68.4	7.70
18	14.46	0.78	68.5	7.70
20	14.44	0.78	68.6	7.70
22	14.48	0.77	68.6	7.70
24	14.43	0.78	68.6	7.69

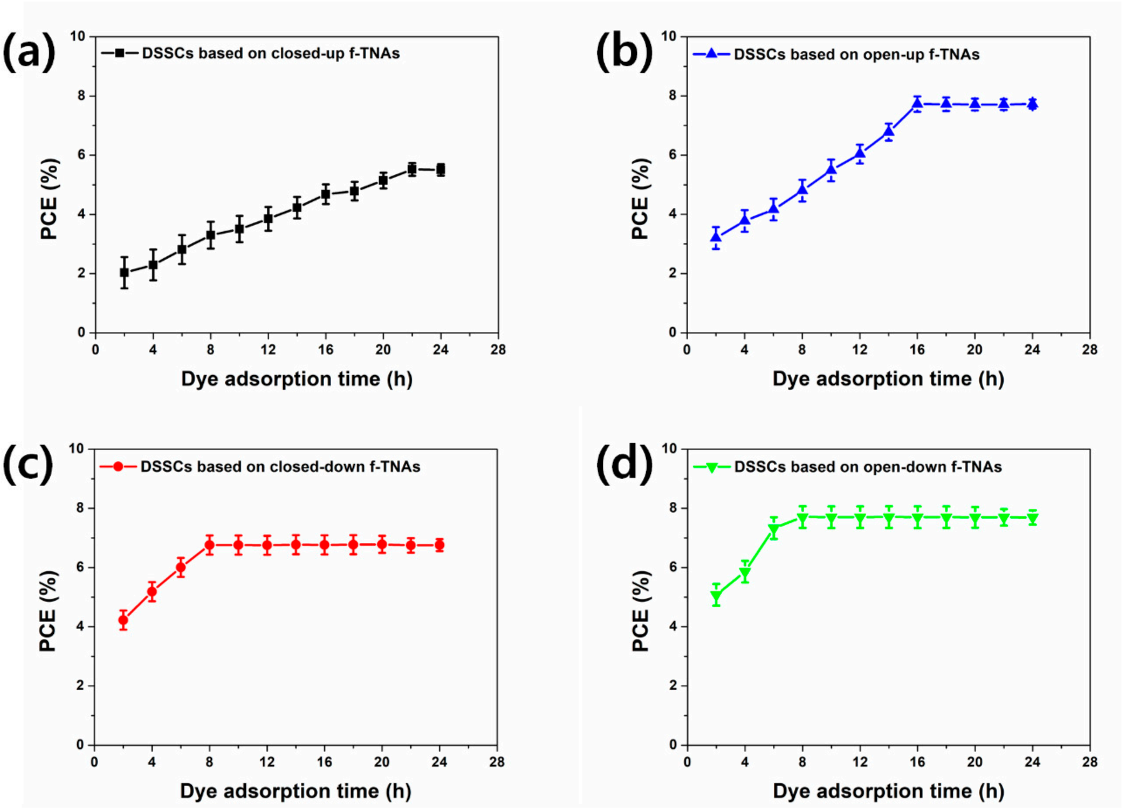

Figure S3. Power conversion efficiency (PCE, %) of DSSCs as a function of dye adsorption time for (**a**) closed-up, (**b**) open-up, (**c**) closed-down, and (**d**) open-down freestanding TiO_2 nanotube arrays (f-TNAs). Error bars represent standard deviation from replicate measurements.

Table S5. Time-dependent PCE and retention of DSSCs during extended one-sun light-soaking.

DSSCs based on	Initial PCE (%)	PCE after 72 h (%)	PCE after 96 h (%)	PCE after 120 h (%)	Retention (120 h)
closed-up f-TNAs	5.52 ± 0.22	5.10 ± 0.21	4.97 ± 0.19	4.80 ± 0.18	87%
closed-down f-TNAs	6.78 ± 0.28	6.24 ± 0.25	6.01 ± 0.23	5.82 ± 0.22	86%
open-up f-TNAs	7.73 ± 0.15	7.16 ± 0.14	6.95 ± 0.13	6.72 ± 0.12	87%
open-down f-TNAs	7.71 ± 0.36	7.19 ± 0.33	7.01 ± 0.31	6.80 ± 0.30	88%

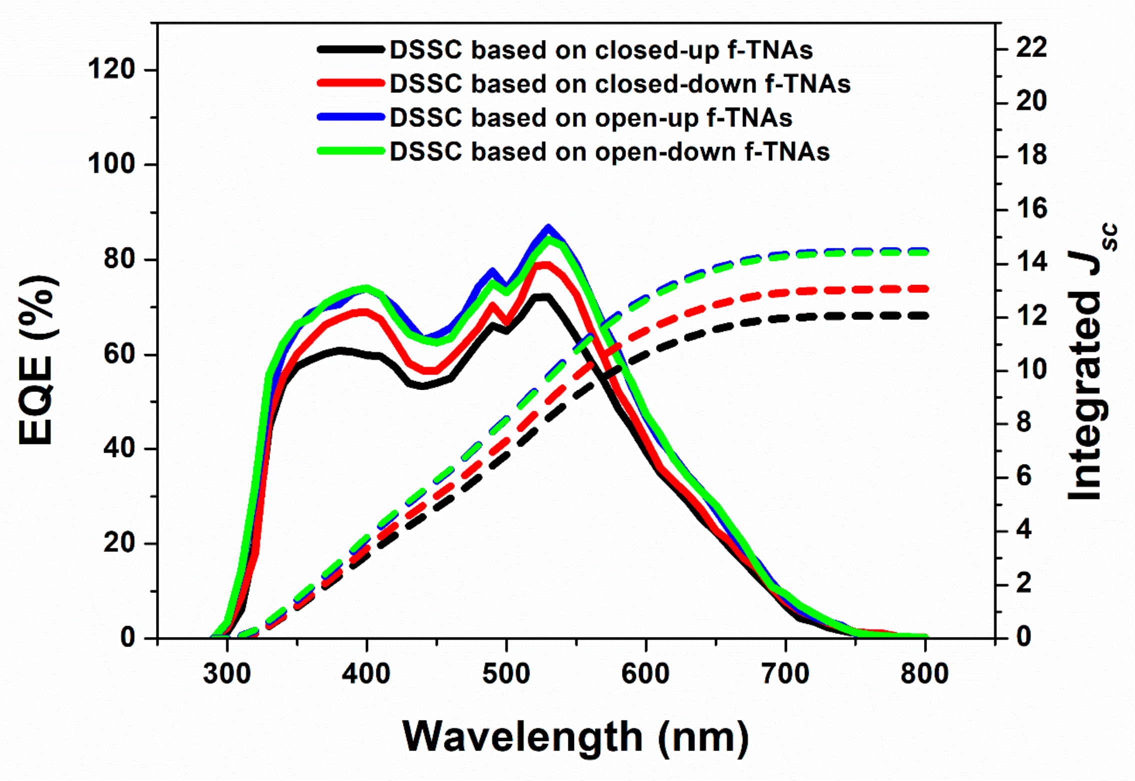

Figure S4. EQE spectra and integrated J_{sc} of DSSCs based on closed-up (black), closed-down (red), open-up (blue), and open-down (green) f-TNAs.

Table S6. Amount of dye loading on closed-up, closed-down, open-up, and open-down f-TNAs by dye adsorption time.

Dye adsorption time (h)	closed-up f-TNAs (nmol/cm^2)	closed-down f-TNAs (nmol/cm^2)	open-up f-TNAs (nmol/cm^2)	open-down f-TNAs (nmol/cm^2)
2	93	159	173	164
4	114	193	186	197
6	132	219	197	221
8	146	229	207	235
10	157	228	217	235
12	167	229	225	234
14	176	230	232	235
16	184	230	238	236
18	191	229	237	234
20	195	229	237	235
22	199	230	238	236
24	200	229	238	237
